# Expression of the repeat genome and aberrant epigenetic factors in cancer

**DOI:** 10.1186/1756-8935-6-S1-P110

**Published:** 2013-04-08

**Authors:** Dawn M Carone, Lisa L Hall, Meg Byron, Jeanne B Lawrence

**Affiliations:** 1 Department of Cell Biology, University of Massachusetts Medical School, Worcester MA, USA

## 

Nearly half of the human genome consists of noncoding repetitive DNA elements, including tandem satellite repeats in large blocks at the pericentric regions of chromosomes and intergenic repetitive elements. While both repeat types were long thought to remain mostly silent, recent evidence indicates that repeats can be expressed, but the extent and regulation of their expression or their potential function(s) remain to be elucidated. Due to their critical location within regions vital for cell division, it is expected that tight regulation of pericentric satellite sequences is essential for both epigenetic and genetic stability. Our data suggests aberrant expression of pericentric satellite RNA is tightly linked to epigenetic misregulation in cancer. It is well known that epigenetic changes can be important in cancer initiation and progression, but studies have focused primarily on the inappropriate silencing and methylation of tumor suppressor genes. While pathologists have long noted the loss of heterochromatic organization in cancer nuclei, and hypomethylation of satellite DNA has been observed, the misregulation of repeat RNAs has only recently been described. Our results provide a link between overexpression of repeat RNAs and aberrant distribution of epigenetic factors in cancer. Our data suggests regulation of the repeat genome has potentially important roles in both normal and neoplastic cells in their ability to affect distribution and recruitment of epigenetic factors.

